# Q344ter Mutation Causes Mislocalization of Rhodopsin Molecules That Are Catalytically Active: A Mouse Model of Q344ter-Induced Retinal Degeneration

**DOI:** 10.1371/journal.pone.0010904

**Published:** 2010-06-02

**Authors:** Francis Concepcion, Jeannie Chen

**Affiliations:** Department of Cell and Neurobiology and Department of Ophthalmology, Zilkha Neurogenetic Institute, Keck School of Medicine, University of Southern California, Los Angeles, California, United States of America; University of Florida, United States of America

## Abstract

Q344ter is a naturally occurring rhodopsin mutation in humans that causes autosomal dominant retinal degeneration through mechanisms that are not fully understood, but are thought to involve an early termination that removed the trafficking signal, QVAPA, leading to its mislocalization in the rod photoreceptor cell. To better understand the disease mechanism(s), transgenic mice that express Q344ter were generated and crossed with rhodopsin knockout mice. Dark-reared Q344ter^rho+/−^ mice exhibited retinal degeneration, demonstrating that rhodopsin mislocalization caused photoreceptor cell death. This degeneration is exacerbated by light-exposure and is correlated with the activation of transducin as well as other G-protein signaling pathways. We observed numerous sub-micrometer sized vesicles in the inter-photoreceptor space of Q344ter^rho+/−^ and Q344ter^rho−/−^ retinas, similar to that seen in another rhodopsin mutant, P347S. Whereas light microscopy failed to reveal outer segment structures in Q344ter^rho−/−^ rods, shortened and disorganized rod outer segment structures were visible using electron microscopy. Thus, some Q344ter molecules trafficked to the outer segment and formed disc structures, albeit inefficiently, in the absence of full length wildtype rhodopsin. These findings helped to establish the *in vivo* role of the QVAPA domain as well as the pathways leading to Q344ter-induced retinal degeneration.

## Introduction

Retinitis pigmentosa (RP) comprises a group of inherited retinal disorders characterized by initial night blindness and a progressive loss of peripheral vision which eventually compromises visual acuity and culminates into total blindness. Epidemiological studies have revealed that RP is heterogeneous both genetically and clinically, and afflicts around 1 in every 3500 to 5000 persons worldwide [1], [2]. The majority of genetic defects causing RP are rod photoreceptor-specific, affecting proteins that include components in the rod phototransduction cascade, structural integrity of rod outer segment (ROS) and vectorial intracellular trafficking. Although in most cases RP is initiated by the death of rod photoreceptors, its progression eventually affects cones, leading to total vision loss. Over 100 different mutations in the rhodopsin (or rod opsin) gene alone have been linked to RP. Moreover, nearly all RP-related rhodopsin mutations are autosomal dominant and collectively have accounted for approximately 30% of all ADRP cases [2], [3], [4], [5]. Based on observations when expressed in cultured mammalian cells [293S cells - 6,COS-1 cells - 7], ADRP-related rhodopsin mutations were classified into two main categories: Class I (15%) and Class II (85%). Interestingly, Class I mutants have no obvious defective biochemical traits, for they closely resembled wild-type (WT) rhodopsin in terms of expression levels, regeneration by binding to 11-cis retinal, and localization to the plasma membrane. On the other hand, Class II mutants have characteristics distinct from WT rhodopsin: their expression levels were markedly lowered; they failed to or poorly regenerated with 11-cis retinal; and in varying degrees they were retained in the endoplasmic reticulum (ER). These empirical properties were attributed to protein mis-folding [6], [7], [8], [9].

The lack of significant biochemical abnormalities in Class I mutants when expressed in cultured mammalian cells indicates that these rhodopsin mutants are properly folded and capable of forming a light-absorbing pigment [6], [7], [8], [9]. Further investigations have revealed that the majority of Class I mutants are clustered at the rhodopsin carboxyl-terminus (C-terminus). Q344ter is such a rhodopsin mutation that causes a severe form of ADRP. In the Q344ter rhodopsin mutant, codon 344, which normally encodes for glutamine, is converted into an early stop codon, thereby resulting in the absence of the QVAPA domain. These five amino acids have been shown to be the minimal sorting signal for the proper budding and trafficking of rhodopsin-bearing transport carriers (RTCs) in a retinal cell-free assay [10], [11]. The role of the QVAPA domain in polarized transport of rhodopsin was also investigated in a previous study using transgenic mice that expressed Q344ter [12]. Expressed Q344ter gave rise to largely normal light responses, indicating that they are properly folded and functional. However, Q344ter caused varying rates of retinal degeneration that correlated with the level of transgene expression. In addition, rhodopsin molecules were not only observed in the ROS but also mislocalized in the rod inner segment and outer nuclear layer. This study showed the importance of the QVAPA domain in the polarized transport of rhodopsin *in vivo*. However, whether cell death was caused directly by rhodopsin mis-trafficking was still not entirely clear, inasmuch as Q344ter was expressed along with endogenous rhodopsin, and it was noted that over-expression of rhodopsin alone can cause retinal degeneration [12], [13], [14]. It was also not clear whether Q344ter alone can transport to the outer segment in the absence of endogenous rhodopsin; a previous morphological study of retinas from S334ter^rho−/−^ mice using light microscopy showed an absence of outer segment structures [15]. Another variable is that the Q344ter mice were not dark-reared. Consequently, the role of rhodopsin mis-trafficking and the added effect of light-exposure was not independently assessed. We addressed these questions by 1) breeding the Q344ter-expressing mice into the endogenous rhodopsin (rho)+/− and rho−/− backgrounds and 2) assessing the extent of retinal degeneration in dark-reared Q344ter mice and comparing this to the effect of controlled light-exposure to retinal degeneration. Consistent with a previous report, we observed that Q344ter^rho+/−^ retinas formed outer segments and exhibited rhodopsin mislocalization to the inner segment and outer nuclear layer compartments [12]. Outer segment structures were not evident in the Q344ter^rho−/−^ retinas by light microscopy or immunohistochemistry on frozen sections. Electron microscopy (EM) showed numerous sub-micron sized vesicles in the subretinal space of Q344ter^rho+/−^ and Q344ter^rho−/−^ retinas. Interestingly, EM revealed outer segment structures with stacked discs in the Q344ter^rho−/−^ retinas. In addition, we provided biochemical evidence for the light-activation of mislocalized Q344ter through light-induced rhodopsin phosphorylation and G-protein activation. Revealing this capability of mislocalized yet properly folded rhodopsin molecules may provide an insight towards discovering a potential cell signaling cascade that triggers rod cell death.

## Results

### Generation of transgenic Q344ter rhodopsin mutant (Q344ter) mice and quantification of transgene expression by RT-PCR and Western blot

**Figure 1:**
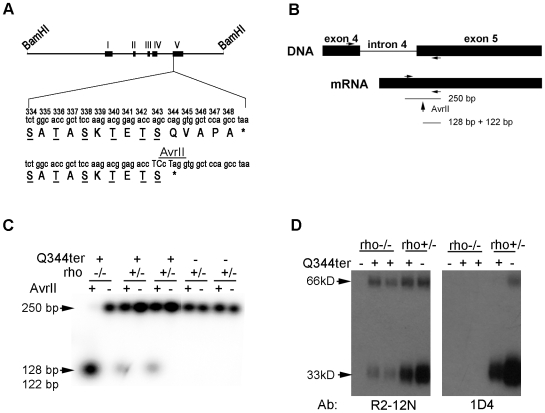
Generation of Q344ter transgenic mice. (**A**) Construct used to generate the Q344ter transgenic mice. In an 11-kb BamHI-flanked genomic clone containing the murine rod opsin gene, codon Q344 was mutated to an early stop signal (bottom *). The resulting rhodopsin mutant is missing the QVAPA domain but retains the six known potential phosphorylation sites (underlined). An AvrII site within the Q344ter transgene was generated by two silent mutations. Capitalized nucleotides denote the introduced point mutations. (**B**) Scheme to establish transcript expression level of the Q344ter transgene. The AvrII site is used to differentiate between Q344ter transgenic and WT transcript species. (**C**) Phosphor-image used to establish transgene-to-total rhodopsin transcript ratio. Total rhodopsin transcripts from each murine retina were amplified by RT-PCR and divided into two equal fractions. After AvrII digestion of one fraction, the enzyme-resistant 250 bp band is compared to the corresponding band from the undigested fraction. (**D**) Detection of the Q344ter mutant by western blot. Equal fraction (1/800) of a retina was loaded onto each lane. R2-12N monoclonal antibody recognizes the amino-terminus and identifies endogenous and Q344ter rhodopsin, while the 1D4 antibody recognizes only full length endogenous rhodopsin. Q344ter expression in rho−/− background was confirmed by R2-12N (left panel). As expected, these species were not detected by 1D4 (right panel). Monomeric and dimeric rhodopsin migrate at 33 kD and 66 kD, respectively.

The Q344ter transgene construct was created by introducing a stop codon into an 11 kb mouse genomic fragment comprising the whole coding sequence of rod opsin and its upstream regulatory regions [16] (Fig. 1A). Therefore, the last five amino acids, QVAPA, are absent from this rhodopsin mutant, while the six Ser and Thr sites that are the substrates of rhodopsin kinase (RK) [17] are retained.

Q344ter transgenic mice were generated and mated with rhodopsin knockout (rho−/−) mice [18] to produce Q344ter transgenic mice with either rho+/− or rho−/− genetic background (Q344ter^rho+/−^ and Q344ter^rho−/−^, respectively). The rho+/− background was used to minimize retinal degeneration that may be induced by rhodopsin over-expression [12], [13], [14]. To isolate the effect of rhodopsin mis-trafficking, Q344ter mice and littermate controls were dark-reared except when noted.

**Figure 2:**
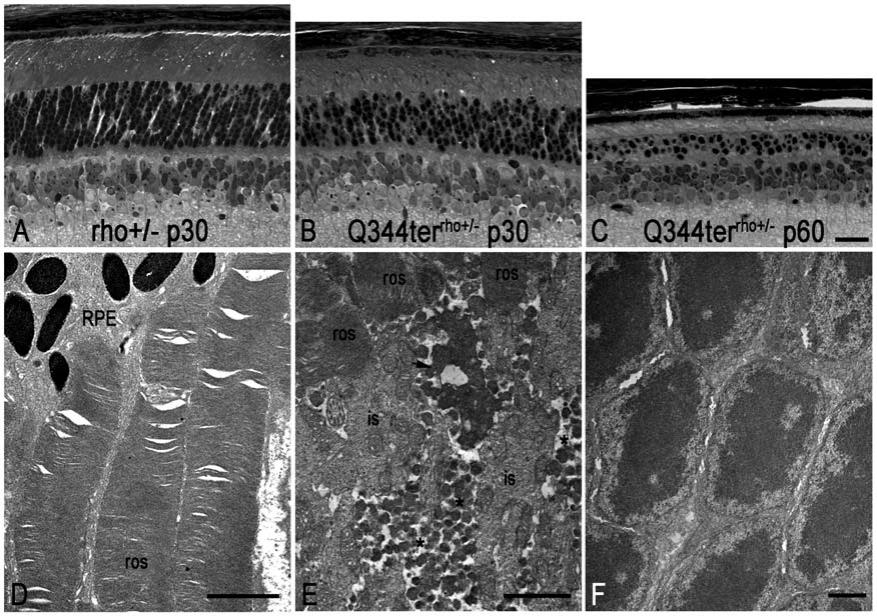
Q344ter transgene causes retinal degeneration independent of light. Images of retinal sections from epoxy-embedded eyecups were taken just above the optic nerve region from Q344ter^rho+/−^ (**B, C**) and their transgene-negative littermate control (**A**) mice at the indicated ages. All mice were born and reared in the dark. (**D**) Rod outer segment structure from control transgene-negative rho+/− mice. (**E**) Vesicular structures (asterisks) within the interphotoreceptor space of Q344ter^rho+/−^ retina. Arrow points to a degenerating structure. (**F**) Outer nuclear layer of Q344ter^rho+/−^ retina is devoid of vesicles. Scale bar in C (20 µm) is also representative for panels A and B. Scale bar  = 1 µm for D, E, and F. ros, rod outer segment; RPE, retinal pigmented epithelium.

The level of Q344ter transgene expression was quantified by RT- PCR. Total RNA was isolated from the retinas of dark-reared transgenic Q344ter mice and their negative littermate controls in rho+/− and rho−/− backgrounds at p28-p30. Transcripts were reverse-transcribed into cDNA followed by PCR (RT-PCR) using a pair of primers mapping to exon 4 and 5 to amplify a 250 bp fragment common to both WT and Q344ter rod opsin transcripts (Fig. 1B). The Q344ter PCR product can be distinguished from the WT by AvrII digestion, which cuts specifically the amplified Q344ter transgene product into 122 bp and 128 bp fragments. Because the PCR primers amplify both WT and Q344ter with equal efficiency, the relative proportion of Q344ter transcript in the total the fraction can be quantitatively obtained. To determine transgene expression levels, we compared intensities between the AvrII-resistant fragment and the same-sized fragment in the mock-digested fraction. As expected, almost the entire amplified PCR product from the Q344ter^rho−/−^ retinas was cleaved by AvrII, while a proportion of the total PCR product from Q344ter^rho+/−^ samples was cleaved by AvrII, and none was cleaved in the transgene-negative samples (Fig. 1C). From the reduction of intensity of the AvrII digested sample as compared to the total undigested sample, we deduced that the level of transgenic gene expression in Q344ter^rho+/−^ retinas to be 24% of total rod opsin transcripts. While a reduction in rhodopsin expression in the rho+/− mice appears to be well tolerated [18] (Fig. 2, A&C), rhodopsin over-expression causes photoreceptor cell death in transgenic mice in a dose dependent manner [12], [13], [14]. Inasmuch as the rhodopsin level in the Q344ter^rho+/−^ retinas lies between that of rho+/+ mice and rho+/− mice, it is not expected to be a contributing factor to retinal degeneration in the Q344ter^rho+/−^ retinas.

At the protein level, it is difficult to distinguish between WT rhodopsin and Q344ter by their size difference. We therefore relied on two mouse monoclonal anti-rhodopsin antibodies: R2-12N and 1D4 to infer expression of Q344ter. R2-12N recognizes rhodopsin's N-terminal region [residues 2–12] [19], and therefore binds to both WT rhodopsin and Q344ter; whereas 1D4 recognizes rhodopsin's C-terminal region [residues 340–348] [20], and therefore binds to endogenous rhodopsin but not to Q344ter. Accordingly, Q344ter is detected by R2-12N but not 1D4 when expressed alone (Fig. 1D, rhodopsin monomer and dimer migrate at 33 kD and 66 kD, respectively). In retinal homogenates from Q344ter^rho+/−^ and transgene-negative littermate controls, rhodopsin was detected by both R2-12N and 1D4 antibodies (Fig. 1D). Here, rhodopsin content appeared to be lower in Q344ter^rho+/−^ retinas than rho+/− retinas despite loading equal amounts of retinal homogenates, suggesting the occurrence of degeneration in these transgenic Q344ter retinas (see below).

### Defective rhodopsin trafficking in the Q344ter transgenic mice causes photoreceptor cell death

To isolate the effect of rhodopsin mis-trafficking on retinal degeneration, Q344ter^rho+/−^ mice and their transgene-negative littermate controls were born and reared in the dark. Retinal morphology of these mice was examined at postnatal day 30 (p30) and p60 (Fig. 2). At p30 the transgene-negative rho+/− littermates exhibit normal retinal architecture with 10–12 layers of photoreceptor cell nuclei, organized outer segments and regularly stacked discs (Fig. 2A). This morphology was maintained at p60 (data not shown). In contrast, the outer nuclear layer thickness was reduced and the outer segments were shortened in Q344ter^rho+/−^ retinas at p30 (Fig. 2B). By p60 the outer segments have disappeared and the outer nuclear layer had thinned to 2–3 cell layers (Fig. 2C). At the ultrastructural level the outer segments of transgene-negative rho+/− mice at p30 showed organized stacks of disc membranes (Fig. 2D). These structures appeared less organized in the Q344ter^rho+/−^ mice (Fig. 2E). In addition, numerous small vesicles that range from 100 to 200 nm in diameter were seen in the extracellular space, or interphotoreceptor space (Fig. 2E, asterisks). The origin of these vesicles is unknown, although their proximity and similarity in electron density suggest that they may be formed from degenerating cellular structures (Fig. 2E, arrow). No vesicles were observed in the outer nuclear layer compartment proximal to the outer limiting membrane (Fig. 2F). The vesicles are reminiscent of that observed by Li et al. in retinas of transgenic mice that express the rhodopsin mutant P347S [21].

**Figure 3:**
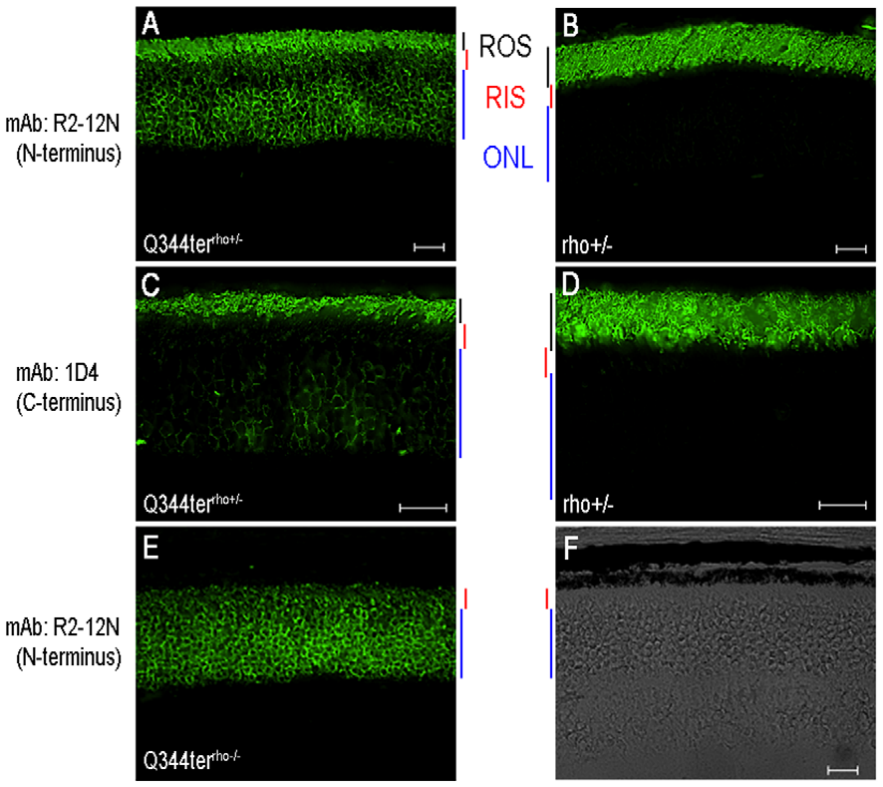
Q344ter rhodopsin is mislocalized and does not support ROS formation when expressed in the absence of endogenous rhodopsin. Q344ter^rho+/−^, Q344^rho−/−^ mice and their transgene negative littermate were dark-reared and sacrificed at p28–31. Frozen retinal sections immunostained with either the anti-N-terminal R2-12N (**A, B, E**), or the anti-C-terminal 1D4 (**C, D**) monoclonal antibodies against rhodopsin. R2-12N immunostaining was restricted to the rod outer segment (ROS) in control rho+/− sections (B), but extended to the rod inner segment (RIS) and outer nuclear layer (ONL) in Q344ter^rho+/−^ sections (A). 1D4 immunostaining revealed the presence of endogenous rhodopsin in the ONL in Q344ter^rho+/−^ retinas (C), which was not observed in retinas from negative transgene littermate controls (D). ROS structures were not detected in Q344ter^rho−/−^ retinal sections when stained with R2-12N (E) or imaged by DIC microscopy (**F**). Scale bars  = 20 µm.

Rhodopsin localization was assessed on frozen retinal sections (Fig. 3). Rhodopsin immunoreactivity is normally localized predominantly in the outer segment, as can be seen in the transgene-negative rho+/− controls (Fig. 3, B&D). However, rhodopsin reactivity extended to the outer nuclear layer (ONL) and inner segment when Q344ter was expressed (Fig. 3A). Q344ter^rho+/−^ retinal sections were also reacted with 1D4, the epitope of which has been mapped to the last nine residues at the carboxyl-terminus of rhodopsin (^340^TETSQVAPA) [20], and therefore recognizes only the full length endogenous rhodopsin (Fig. 3C). The weaker signal in the ONL seen in the 1D4 section shows that some endogenous rhodopsin molecules were present in this cellular compartment. However, the majority of the signal in the outer nuclear layer in Fig. 3A appears to be due to Q344ter. Together, the data presented so far indicate that Q344ter mislocalization causes retinal degeneration in the mammalian retina.

**Figure 4:**
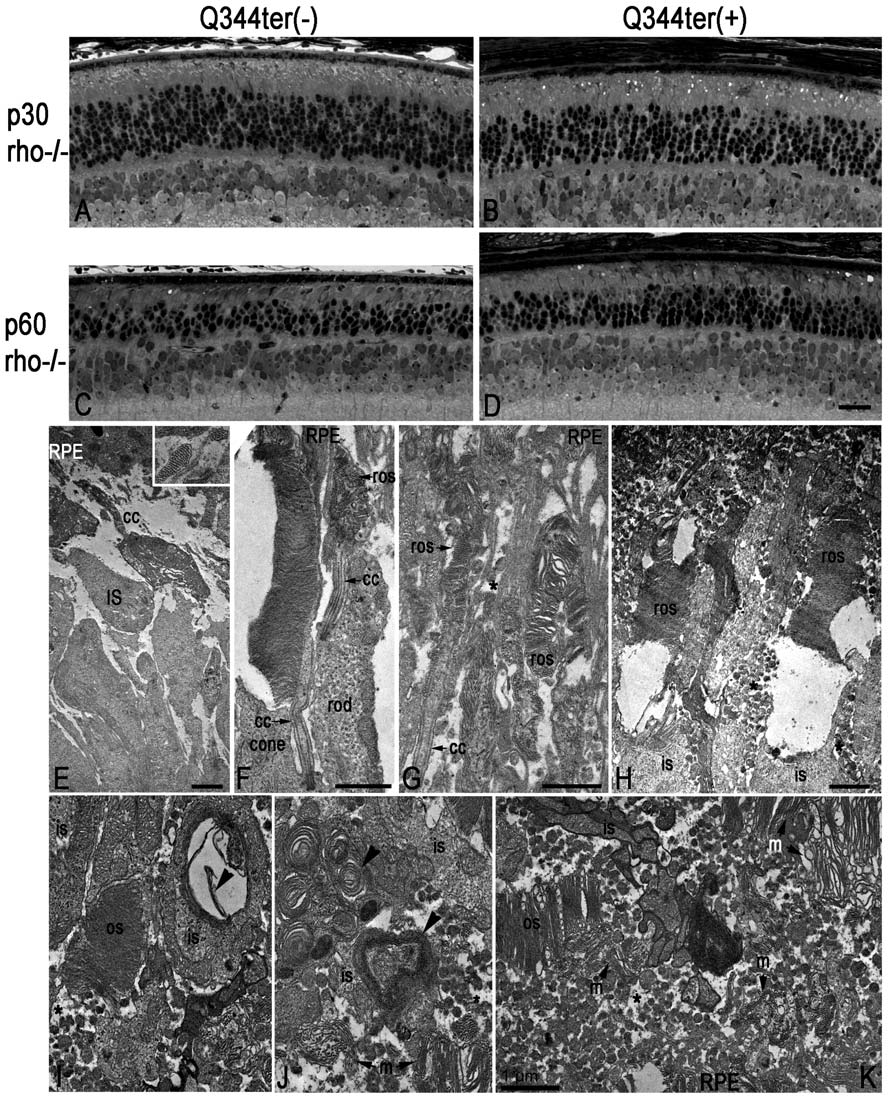
Q344ter transgene does not accelerate retinal degeneration in rho−/− retinas. As in Figure 2, these images of retinal sections from epoxy-embedded eyecups were taken just above the optic nerve region from Q344ter^rho−/−^ (**B, D**) and their transgene-negative littermate control (**A, C**) mice at p30 and p60. At both time points, ONL thicknesses appear similar in Q344ter^rho−/−^ mice and their littermate controls (compare A to B and C to D). Although progressive ONL thinning was observed in both groups, Q344ter does not appear to accelerate degeneration already occurring in rho−/− mice. Scale bar in D (20 µm) is representative for panels A–D. (**E**) Rho−/− rod photoreceptors do not elaborate outer segment structures. Instead, membrane tubules are seen (inset). The subretinal space is devoid of vesicular structures. Panels **F**–**H** are from p30 Q344ter^rho−/−^ retinas, and **I**–**K** are from p60 Q344ter^rho−/−^ retinas. (**F**) Short and disorganized rod outer segment can be seen distal to the connecting cilia. A neighboring cone photoreceptor with a much more intact outer segment is shown for comparison. (**G**) The rod outer segments shown here are thinner than normal (compare with Figure 2D). These structures are surrounded by apical processes from the RPE. (**H**) The membranous discs within some outer segment structures appear to be unstable. Numerous vesicular structures are present in the extracellular space (asterisks). (**I-K**) Vesicular structures are present in the interphotoreceptor space of Q344ter^rho−/−^ retinas. Outer segments containing discs are evident, but they are significantly compromised both in size and organization. Scale bars for E-K = 1 µm. Panels I-K are taken at the same magnification. os, outer segment; ros, rod outer segment; is, inner segment; cc, connecting cilium; RPE, retinal pigmented epithelium; m, membranous debris.

**Figure 5:**
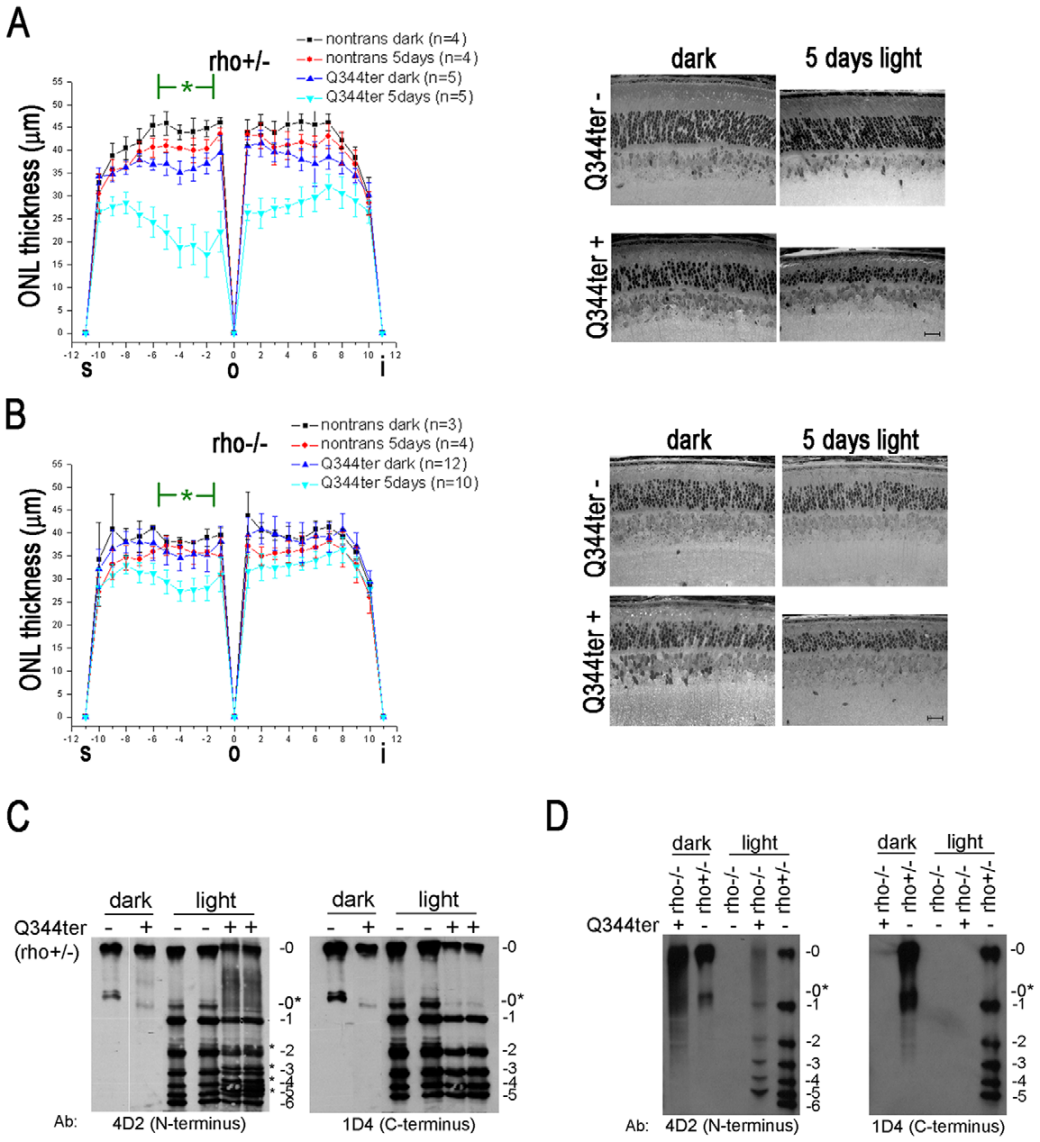
Light exacerbates Q344ter-induced retinal degeneration and activates Q344ter in the inner segment and outer nuclear layer. (**A**) Q344ter^rho+/−^ and nontransgenic littermate control mice or (**B**) Q344ter^rho−/−^ and nontransgenic littermates control mice that were either dark-reared only or exposed to continuous light (3000 lux with undilated pupils) for five days were sacrificed at p28–31. Retinal sections near the optic nerve were analyzed by retinal morphometry. The diagram displays the mean (± SD) ONL thickness along the entire span of the retina. We focused on a light-sensitive region in the superior half near the optic nerve marked by a green asterisk where a slight ONL thinning occurred in light-exposed nontransgenic mice when compared to their dark-reared counterparts. Under dark-rearing, Q344ter retinas showed a moderate level of degeneration when compared to their transgene-negative littermate controls (p≤0.05). Light-exposure induced a severe form of degeneration in Q344ter transgenic retinas when compared to both light-exposed nontransgenic retinas and dark-reared Q344ter transgenic retinas (p≤0.05). A representative light microscopy image within this region from each group is displayed to the right. Scale bar  = 20 µm. (**C**) Isoelectric focusing gel of retinal extract from designated mice was blotted onto nitrocellulose and probed with the indicated antibodies against rhodopsin. The numbers to the right of each membrane image corresponds to the number of phosphates. In the left panel both Q344ter and WT rhodopsin molecules are detected by the anti-N-terminal rhodopsin mAb 4D2. Only non-phosphorylated rhodopsin (0) and apo-opsin (0*) species were detected in retinas from dark-reared mice. Light-exposure produced multiple phosphorylated rhodopsin species. Moreover, four extra bands (*) were detected in light-exposed Q344ter^rho+/−^ retinas. In the right panel, only WT rhodopsin molecules are detected by the anti-C-terminal rhodopsin mAb 1D4. Note that the four extra bands in the light-exposed Q344ter^rho+/−^ retinas detected by 4D2 are absent. (**D**) Mislocalized Q344ter molecules undergo light-dependent phosphorylation. With the rho−/− background, Q344ter molecules are mislocalized as shown by the absence of apparent ROS structures with R2-12N immunostaining (Fig. 3E). Retinal homogenates from light-exposed Q344ter^rho−/−^ mice were examined under similar IEF conditions described in (B). Light-dependent phosphorylation patterns of Q344ter molecules were detected by 4D2 (left panel) but not by 1D4 (right panel). This light-dependent phosphorylation pattern of Q344ter in these mice show that mislocalized Q344ter is capable of light-activation.

Q344ter can be co-transported on the post-Golgi vesicles to the outer segment along with the full length endogenous rhodopsin [12]. We bred Q344ter into the rho−/− background to see whether it may traffic alone to the outer segment, thereby revealing an alternate trafficking motif. In the Q344ter^rho−/−^ frozen retinal sections, rhodopsin immunofluorescence was seen in the outer nuclear layer and inner segment (Fig. 3E). The outer segment layer is not detectable either by rhodopsin immunofluorescence or in the DIC image (Fig. 3, E&F). Similarly, light micrographs of epoxy resin embedded sections failed to show outer segment structures in Q344ter^rho−/−^ retinas. Their morphology was indistinguishable from their transgene-negative littermates at p30 (Fig. 4, A&B) or p60 (Fig. 4, C&D). Interestingly, the degree of retinal degeneration was similar at p30 and p60 in the presence or absence of Q344ter, indicating that Q344ter mis-trafficking does not accelerate the rate of retinal degeneration in the rhodopsin knockout (rho−/−) background. Quantitative measurements of outer nuclear layer thickness of retinas from dark-reared Q344ter^rho−/−^ and their transgene-negative littermates at p30 are shown in Fig. 5B, blue and black traces, respectively.

At the ultrastructural level, rods in the rho−/− retinas do not elaborate outer segments (Fig. 4E), consistent with previous reports [18], [22], [23]. In these samples membranous stacked tubules of ∼15 nm diameter were observed in cross sections and tangential sections (Fig. 4E, inset). These may represent membrane cargoes that failed to form discs in the absence of rhodopsin. Unexpectedly, outer segment structures were revealed under EM in the retinas of Q344ter^rho−/−^ mice at p30 (Fig. 4, F–H). In some instances shortened and disorganized outer segment structures (ros) can be seen distal to the connecting cilia (cc, Fig. 4, F&G). It is not clear whether these disorganized structures result from a defect in outer segment formation or stability. A similar morphologic pattern was seen in the Q344ter^rho−/−^ retinas at p60 (Fig. 4, I–K). Inclusions of membranous whirls can be seen in some inner segments (Fig. 4, I&J, arrowheads), and abundant membranous debris is present in the subretinal space. Some of this debris are in the form of discs, while others appear to be tubules similar to that seen in the rho−/− retinas (Fig. 4, J&K, m:arrows). At both ages abundant 100–200 nm diameter vesicular structures were observed in the interphotoreceptor space (Fig. 4, G–K, asterisks). Thus, Q344ter is capable of forming outer segment structures, although the morphology of these structures suggests that they are not structurally stable.

### Q344ter-initiated retinal degeneration is accelerated by light-exposure

The heterogeneous disease progressions among ADRP patients inheriting the same rhodopsin mutation indicate that environmental conditions contribute to the severity of this disease. One potential environmental candidate is light-exposure. By rearing Q344ter mice and their negative littermate controls under constant darkness versus light- exposure (3000 lux constant light-exposure for 5 days), we could isolate the effect of rhodopsin mis-trafficking alone as well as the combined effect of mis-trafficking and light-exposure on retinal degeneration.

We measured the degree of degeneration by retinal morphometry based on a previous method [24]. For statistical analysis between sample populations, ONL thickness measurements were recorded within a sub-region of the superior half and near the optic nerve of each retina (Fig. 5A), which previously was revealed to be the most sensitive region to light-damage [24], [25]. Control pigmented mice of the C57/B6 genetic background with undilated pupils do not undergo light-damage under our experimental protocol [26]. In some experiments, we observed a small effect of light-exposure in control rho+/− retinas (Fig. 5A). As mentioned previously, dark-reared Q344ter^rho+/−^ retinas had thinner ONL than their non-transgenic littermates, an effect attributable to rhodopsin mislocalization (Fig. 5A). This degeneration is quite profoundly exacerbated by light-exposure (Fig. 5A). In contrast, rho−/− retinas did not exhibit light-damage (Fig. 5B), consistent with a previous report that rhodopsin is required for light-induced photoreceptor cell death [27]. Interestingly, outer nuclear layer thicknesses were similar in dark-reared Q344ter^rho−/−^ mice and their trangene-negative littermates (Fig. 5B, blue and black traces, respectively). Perhaps the deleterious effect of rhodopsin mis-trafficking is offset by the beneficial effect of rhodopsin expression in the rho−/− retina. This effect is maintained through p60 (Fig. 4). On the other hand, light-exposure also exacerbated retinal degeneration in Q344ter^rho−/−^ mice (Fig. 5B), suggesting that mislocalized Q344ter is activated by light-exposure.

### Mislocalized rhodopsin is capable of light-activation

Exacerbation of retinal degeneration by light-exposure has been observed for rhodopsin mutants that may or may not initiate a cell death cascade that involves phototransduction. In a previous study involving cultured rod cells, it was demonstrated that mislocalized rhodopsin could be activated and leads to cell death [28]. Whether mislocalized rhodopsin is capable of light-activation *in vivo* in the vertebrate retina has not been addressed. Substantiating this property may provide an important first step towards discovering the mechanism(s) that leads to the observed light-accelerated retinal degeneration in our transgenic Q344ter mouse model as well as other Class I rhodopsin mutants.

We examined whether mislocalized rhodopsin is capable of light-activation by assessing the light-dependent rhodopsin phosphorylation by rhodopsin kinase [29], [30]. Phosphorylated rhodopsin species can be separated by isoelectric focusing (IEF), blotted onto nitrocellulose, and visualized using antibodies against rhodopsin. As shown in Fig. 5C, only non-phosphorylated rhodopsin and opsin molecules were detected in retinas from dark-reared mice. These non-phosphorylated species also existed in light-exposed retinas, which indicate that not all rhodopsin molecules in the retinas are phosphorylated under the described light conditions. Upon light-exposure, six phosphorylated rhodopsin species that can be visualized with 4D2, an antibody against rhodopsin's N-terminus [31], appeared in the rho+/− retinas (Fig. 5C, middle lanes). These species are also present in Q344ter^rho+/−^ retinas, but four additional bands are also present in these samples (Fig. 5C left panel, 4D2, two lanes on the right). To confirm that these four additional species originate from the Q344ter population, we subsequently probed the same membrane with 1D4 (Fig. 5C right panel, 1D4). We reasoned that if the four additional rhodopsin species are phosphorylated Q344ter molecules, then they would not be recognized by 1D4. Indeed this was found to be the case: the bands recognized by 1D4 are the same between rho+/− and Q344ter^rho+/−^ samples, corresponding only to full length endogenous rhodopsin. Interestingly, 1D4 did not recognize the hexa-phosphorylated rhodopsin species in both retinal types. Based on a previous study that mapped the residues that contribute to 1D4 binding, it is likely that phosphorylation at the T342 position abolished the 1D4 epitope [20].

Because Q344ter can be co-trafficked with full length rhodopsin to the outer segment, the above experiment does not fully address whether Q344ter located to the outer nuclear layer and inner segment is capable of light-activation. To address this question directly, phosphorylation of Q344ter was assessed in the rho−/− background in which the majority of Q344ter is located in the outer nuclear layer and inner segment compartments (Fig. 3E). As can be seen in Fig. 5D, non-phosphorylated Q344ter^rho−/−^ in the dark-adapted sample is recognized by 4D2 but not 1D4. Interestingly, 4D2 identified five phosphorylated Q344ter species upon light-exposure as opposed to six in the rho+/− control sample. We speculate that the Q344ter truncation affected the ability of rhodopsin kinase to phosphorylate Ser343, which is the carboxyl-terminal residue in Q344ter. As expected, these phosphorylated Q344ter species are not recognized by 1D4. These results indicate that mislocalized Q344ter is capable of light-activation.

### Transducin signaling contributes to retinal degeneration in transgenic Q344ter mice

**Figure 6:**
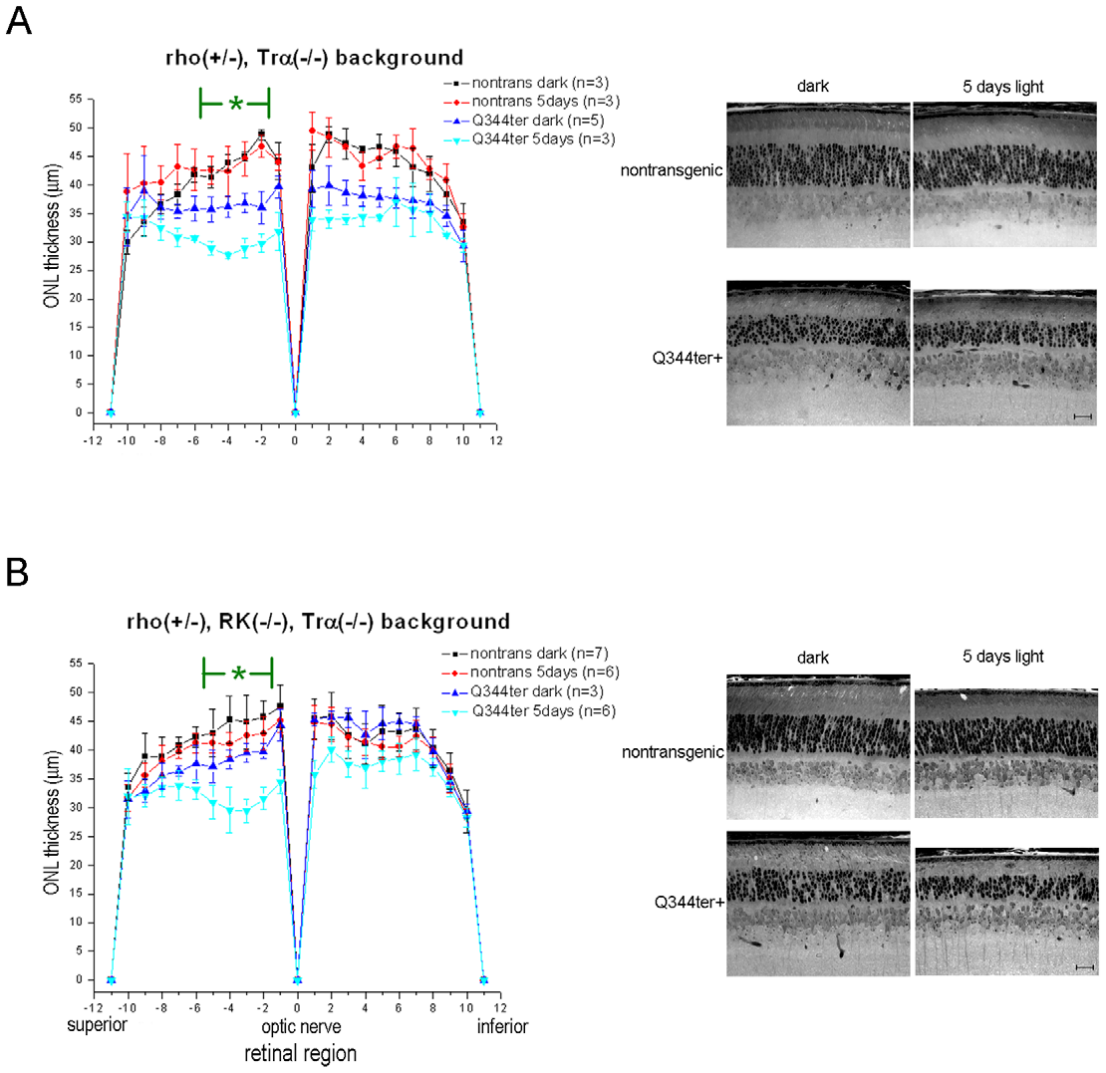
Light-exacerbated retinal degeneration in Q344ter mice is ameliorated in the Trα−/− background. Dark-reared mice were kept in darkness or exposed to light for 5 days as in Fig. 5 (**A**) Retinal morphometry of Q344ter^rho+/−^ in the Trα−/− background. In the dark-reared group expression of the Q344ter transgene consistently caused a moderate thinning of the ONL. Light-exposure had no noticeable effect on the transgene negative mice, but caused further thinning of the ONL in Q344ter^rho+/−^ mice. However, this effect was less severe when compared to the Trα+/+ background (Fig. 5A). (**B**) The Q344ter^rho+/−^, Trα−/− mice were further crossed into the RK−/− background to prevent light-induced formation of stable rhodopsin/Arr1 complexes. No additional rescuing effect beyond that seen in the Trα−/− background was observed in the light-exposed transgenic Q344ter mice in the RK−/−, Trα−/− background. Representative light micrographs of retinal sections are shown on the right. Scale bars  = 20 µm.

Since Q344ter is capable of activating transducin [12], Q344ter^rho+/−^ mice were bred into the Trα−/− background to see whether transducin signaling contributed to photoreceptor cell death. Again, dark-reared Q344ter^rho+/−Trα−/−^ mice had thinner outer nuclear layer when compared to nontransgenic littermates (rho+/−, Trα−/−), indicating that retinal degeneration induced by mis-trafficking alone is not dependent on the presence of transducin (Fig. 6A). Light-exposure caused a further thinning of the outer nuclear layer although to a lesser degree when compared to light-exposed Q344ter^rho+/−^ mice that express transducin (compare the cyan traces in Fig. 6A and Fig. 5A). These results suggest that transducin signaling in the outer nuclear layer and inner segment compartment contributed to photoreceptor cell death. The lack of full rescue indicates that additional light-induced pathways are involved.

Light-dependent formation of stable rhodopsin/arrestin (Arr1) complex has been found to be toxic to *Drosophila* photoreceptors [32], [33]. We have also previously demonstrated that the K296E opsin mutant caused retinal degeneration in the mammalian retina through formation of K296E/Arr1 complex [34]. We hypothesized that this effect is due to the mislocalized K296E forming a complex with Arr1 in the outer nuclear layer and inner segment. Since mislocalized Q344ter is capable of light-activation in these compartments, light-dependent formation of Q344ter/Arr1 complex may be an underlying mechanism for light-induced cell death in these mice. Although the genetic background (Q344ter^rho+/−^, Arr1−/−, Trα−/−) would provide a more direct test for potential toxicity arising from mislocalized rhodopsin/Arr1 complexes, we and others have observed evidence for an unknown genetic factor that appears to make a subpopulation of Arr1−/−, Trα−/− mice susceptible to severe light-induced degeneration even at low light intensities [34], [35], making this genetic background unsuitable for the present study. To test the hypothesis, we instead crossed the Q344ter^rho+/−^ mice and their negative littermate controls into the RK−/−, Trα−/− genetic background, which does not appear to exhibit low light-induced retinal degeneration. Removing Trα was necessary when deleting RK, since the absence of RK leads to prolonged activation of the phototransduction cascade in the presence of dim light and will induce retinal degeneration through a different mechanism [35], [36], [37]. In the absence of RK there is no light-induced rhodopsin phosphorylation, and without phosphorylation, arrestin's affinity for light-activated rhodopsin is low [37], [38], [39], [40], and therefore little or no stable complex will be formed. If the rhodopsin/Arr1 complex contributed to the severe retinal degeneration in the light-exposed transgenic Q344ter^rho+/−^ background (Fig. 5A), we would expect an amelioration of the retinal morphology in the light-exposed transgenic retinas when Q344ter is expressed in the RK−/−, Trα−/− genetic background. As shown in Fig. 6B, dark-reared Q344ter mice also had thinner ONL than their non-transgenic littermate controls in the RK−/−, Trα−/− background, indicating that mislocalization of Q344ter alone is deleterious to rod photoreceptors in general. This degeneration is again exacerbated by light-exposure. The degree of degeneration is similar to that in the Trα−/− background, suggesting that the degree of rescue is contributed by removing transducin signaling and not by formation of Q344ter/Arr1 complex in the ONL and inner segment compartments.

### Light-induced G-protein activation visualized by [^35^S]GTPγS autoradiography

**Figure 7:**
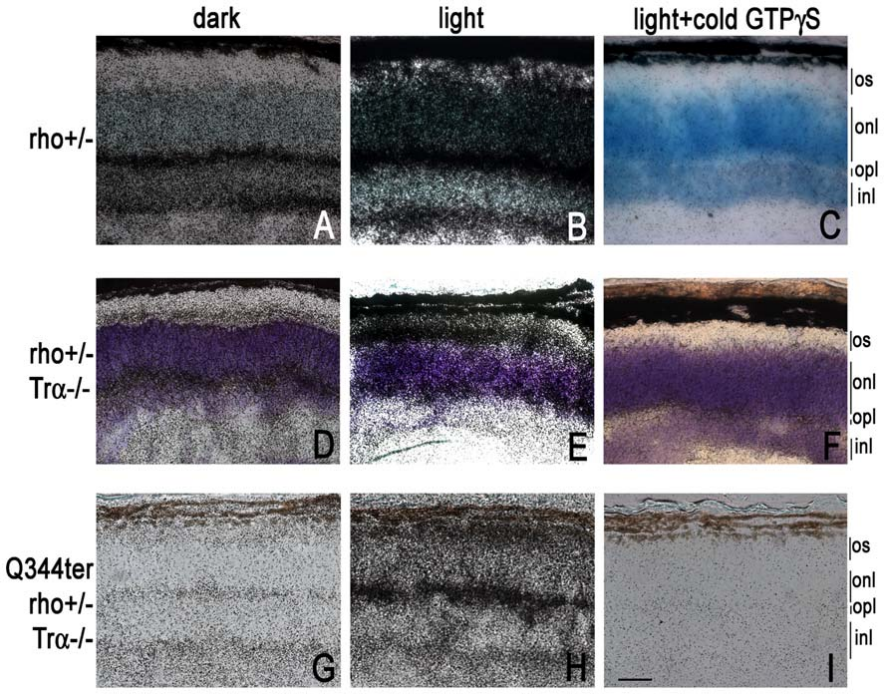
Light-dependent GTPγS loading (20 min exposure) in transgenic Q344ter frozen retinal sections. [^35^S]GTPγS binding *in situ* was performed on unfixed frozen retinal sections from mice with the indicated genetic backgrounds. Basal [^35^S]GTPγS loading in the dark labels the synaptic layers (**A, D, E**), while light-exposure lead to additional labeling at the inner and outer segment compartments (**B, E, H**). Panels **C**, **F,** and **I** show non-specific background labeling. Scale bar  = 20 µm. All panels are taken at same magnification.

Results from Figures 5 and 6 suggest that light-induced retinal degeneration in Q344ter is in part mediated by transducin signaling, and that additional pathway(s) are involved. As a starting point of identifying such pathways, we investigated the participation of G-proteins inasmuch as rhodopsin is a G-protein coupled receptor and is required for light-damage [27]. To visualize activation of G-proteins *in situ*, frozen, unfixed retinal sections from the indicated mice were incubated with [^35^S]GTPγS followed by autoradiography. In the darkness, the outer plexiform layer is consistently labeled, reflecting the involvement of GTP binding proteins in synaptic vesicle trafficking ([41], Fig. 7, A, D, G). In control rho+/− section, the outer segment layer became strongly labeled upon light-exposure due to transducin activation at this location (Fig. 7B). Notably, light also stimulated [^35^S]GTPγS labeling in the outer segment and inner segment layers in the Trα-/− retina, both in the littermate negative control (Fig. 7, D&E) and in Q344ter positive retina (Fig. 7, G&H). The specificity of the [^35^S]GTPγS binding is demonstrated in Fig. 7 (C, F, I) where an excess of cold GTPγS was included in the incubation. Thus, rhodopsin activation catalyzed GTP loading in protein(s) other than it's known *in vivo* target, transducin. Such novel signaling pathway(s) may contribute to light-induced retinal degeneration in these mice.

## Discussion

In this investigation we utilized transgenic Q344ter mice to gain a better understanding of the pathways that contribute to retinal degeneration in ADRP patients inheriting this rod opsin mutation. It is known that the rhodopsin carboxyl-terminus contains a sorting motif which associates with cytoplasmic proteins that assist in its transport from the site of synthesis to the outer segment [42], [43]. *In vitro* evidence, transgenic mouse studies, as well as occurrence of naturally occurring mutations in human populations point to the carboxyl-terminal VxPx motif for interaction with other transport proteins [10], . Consistent with this idea, Q344ter, lacking this motif, exhibit a trafficking defect: immunostaining of Q344ter^rho+/−^ retinal sections with the R2-12N antibody revealed that rhodopsin localized not only to the rod outer segment but also abnormally accumulated in the inner segment and outer nuclear compartments. We show that Q344ter expressed at 24% of total rhodopsin caused a moderate rate of retinal degeneration in dark-reared mice, an experimental condition that isolated the effect of rhodopsin mislocalization (Fig. 2). This finding is consistent with a previous study by Tam et al. that expressed Q350ter (analogous to mammalian Q344ter) in transgenic Xenopus laevis [45]. They found that mislocalized rhodopsin does not require activation to cause retinal degeneration. Interestingly, we observed in a previous study that the rhodopsin mutant, S334ter, did not cause noticeable retinal degeneration in dark-reared mice when it was expressed at 10% of total rhodopsin [15]. Therefore, the photoreceptor cell appears to tolerate some degree of rhodopsin mislocalization by low level expression of these trafficking mutants.

Electron microscopy showed numerous of sub-micron sized vesicles and other debris in the interphotoreceptor matrix of Q344ter^rho+/−^ retinas. The presence of these vesicles was also noted in the P347S transgenic mice [21] as well as in the S334ter^rho+/−^ retinas (data not shown). Thus, accumulation of extracellular vesicles appears to be a commonality for rhodopsin with carboxyl-terminal mutations that affect the QVAPA domain. The presence of these vesicles has also been observed in other mutant mice that include *tulp1* [46], [47], *tubby* [48] and *pcd* [49]. These gene products may participate in the same pathway that regulates rhodopsin transport to outer segment and thus share the same pathogenesis of disease.

Ultrastructural studies of Q344ter^rho−/−^ retinas revealed, for the first time, the presence of outer segment structures which were not detectable under light microscopy. How did the Q344ter molecules arrive at the outer segment? One possibility is the presence of a weak trafficking motif aside from VxPx. Alternatively, these structures may have formed from bulk flow of post-Golgi vesicles that arrived at the apical regions of the rod cell. Future experiments will be needed to address these possibilities. Another novel observation from our EM study is that the outer segment discs from Q344ter^rho−/−^ rods appeared to be disorganized (Fig. 4H). It is tempting to speculate that the QVAPA domain may contribute to the retention of rhodopsin at the outer segment and/or stability of the disc structures. Yet another possibility is that, due to the trafficking defect, the Q344ter molecules did not reach a critical density on the disc membranes to support stable disc structures. This proposed model would be largely consistent with a previous study on another truncated rhodopsin, S334ter [50]. Lee and Flannery showed S334ter molecules at the distal end of connecting cilium when expressed in the absence of endogenous WT rhodopsin. They proposed this localization to be the outcome from random events and not directional movement of the truncated rhodopsin molecules.

Our current study also reveals that light-exposure exacerbated Q344-induced retinal degeneration. This observation is correlated with our novel finding that mislocalized Q344ter is capable of light-activation. These findings allow us to formulate the hypothesis that light-dependent catalytically active Q344ter would become accessible to proteins not normally encountered by rhodopsin and initiate a signaling pathway in the inner segment/outer nuclear compartments that leads to accelerated degeneration. Such examples could be the activation of transducin or an unknown G-protein [28]. The amelioration of the light-induced retinal degeneration in Q344ter^rho+/−^, Trα−/− mice suggests that Q344ter-catalyzed transducin signaling in the inappropriate cellular compartments of outer nuclear layer and inner segment may play a role. Because the GAP complex that deactivates transducin-GTP is localized predominantly in the outer segment [51], [52], activated transducin in the inner segment and outer nuclear layer compartments may remain in the active conformation for a prolonged period of time and cause cell death. Although an improvement of light-induced retinal degeneration was observed in the Trα−/− background, the lack of complete rescue indicate that additional pathways are also involved. We investigated the participation of G-proteins because rhodopsin is a GPCR and is required for light-damage [27]. Indeed, the data from [^35^S]GTPγS labeling showed light-dependent GTP loading in the inner and outer segment compartments in Trα−/− retinas. It is known that rhodopsin activates G_i_ and G_o_ efficiently *in vitro* [53], [54]. Notably, G_i2_ is expressed in the photoreceptors [55]. Therefore, it may be a target for rhodopsin activation *in vivo*. Alternately, rhodopsin activation may indirectly lead to activation of small G-proteins. Additional experiments are required to identify these signaling pathways and their effect in the photoreceptor cell.

We tested the hypothesis that light-activation of Q344ter in the inner segment may have lead to formation of toxic rhodopsin/arrestin complex that signaled cell death. However, a lack of additional rescue in the Q344ter^rho+/−^, Trα−/−, RK−/− mice indicate that this pathway is likely not involved in this mouse model. Such a difference between Q344ter and K296E mutations may lie in the degree of phosphorylation and the stability of the rhodopsin/Arr1 complex: K296E cannot bind 11-cis retinal and exists in an active conformation as it is synthesized [56]. We found it to be hightly phosphorylated and formed a stable complex with Arr1 [34]. Q344ter, on the other hand, becomes phosphorylated upon light-exposure and is only capable of five phosphorylations (Fig. 5B). Unlike K296E, it undergoes MII decay. For these reasons Q344ter may not form a stable complex with Arr1. In summary, these results underscore the complex nature of retinal degeneration induced by rhodopsin mutations.

It was observed that retinal degeneration in P347S transgenic mice is accelerated in the absence of Trα [57]. In that study the mice were raised under cyclic light, making a direct comparison to our study difficult. We observed that Trα had little or no effect on the degree of retinal degeneration in dark-reared Q344ter mice, whereas the absence of Trαwas protective on light-exposed Q344ter mice (Figures 5A and 6). This discrepancy may be due to differences in experimental protocols (cyclic light-reared for P347S vs. dark-reared for Q344ter) or the nature of the rhodopsin mutation itself. In the P347S mice lipofuscin fluorophores were found to be elevated, perhaps due to a faster decay of MII to apo-opsin and free all-trans-retinaldehyde [57]. We did not observe an accumulation of lipofucsin in the Q344ter mice.

Light-exposure has been reported to accelerate retinal degeneration in animal models expressing P23H [25], [58], [59], [60], [61]. In these situations, P23H most likely perturbs processes in rod cells without itself becoming photolyzed since P23H is mis-folded. In this case light-exposure likely compounded the deleterious effect of P23H through a different pathway. Interestingly, studies have shown that patients inheriting Class I rhodopsin mutants, i.e. Q344ter, tend to have more severe cases of ADRP than patients inheriting Class II rhodopsin mutants, i.e. P23H [62], [63]. Because patients are never restricted to dark-only conditions, the above observation may be attributed to the activation of an unknown yet deleterious rhodopsin signaling pathway(s) specific to the mislocalized Class I rhodopsin mutants. This hypothesis is rendered plausible with our observation that mis-trafficked Q344ter molecules are capable of light-excitation.

## Materials and Methods

### Ethics Statement

All experimental procedures were performed in accordance with the regulations established by the National Institute of Health as well as with the Association for Research in Vision and Ophthalmology. The experiments involving vertebrate animals have been approved by the University of Southern California Institutional Animal Care and Use Committee (IACUC, Protocol #10275).

### Generation of transgenic Q344ter mice and genotype analysis by PCR and Southern blotting

The Q344ter rod opsin mutation, along with two silent mutations which generated an AvrII restriction site designed for genotyping purposes, was introduced into an 11 kb BamHI-flanked genomic clone of the murine opsin gene [16]. The construct was purified by the CsCl_2_ gradient method, and the mutated rod opsin gene was released from its vector by BamHI digestion. The digested DNA fragments were separated in a 0.8% agarose gel, and the BamHI-flanked Q344ter gene fragment was gel-extracted by using the QIAEXII kit (Qiagen, Valencia, CA). After further purification with an Elutip-D column (Whatman Schleicher & Schuell, Sanford, ME), this DNA fragment was microinjected into fertilized eggs of donor B6D2F1 females to generate transgenic Q344ter mice (Norris Transgenic Core facility, Keck School of Medicine of USC, Los Angeles, CA). All transgenic Q344ter mice and their negative littermate controls were dark-reared (except when noted) to prevent potential undesired light-dependent retinal degeneration [60], [64], [65], [66], and to isolate the effect of Q344ter mis-trafficking to photoreceptor cell death.

For genotyping, mouse-tail biopsy samples were used to extract genomic DNA, from which a 376 bp PCR product was generated by using the primer pair FACmRho6020 (5′TCCGGAACTGTATGCTCACCAC3′) and Rho3 (5′TGAGGGAGGGGTACAGATCC3′). This amplified product then was digested by AvrII. If the mouse possessed the Q344ter transgene, two fragments (92 bp and 284 bp) would result.

Q344ter transgenic mice were bred to rho−/− mice [18] to generate transgenic mice with the endogenous rhodopsin +/− and −/− genetic background (rho+/− and rho−/−, respectively). PCR was performed to detect the presence of the rod opsin null allele by using primers Rh1.1 (5′ GTGCCTGGAGTTGCGCTGTGGG3′ ) and Neo3 (5′ CGGTGGATGTGGAATGTGTGCGAG 3′). To distinguish between hemizygous (+/−) and homozygous (−/−) rhodopsin knockout mice, we performed Southern blot analysis based on a previous protocol [67].

### Determination of transgene expression level by RT-PCR

Q344ter transgene expression level was determined by quantifying the mutant-to-total transcript ratio in transgenic Q344ter^rho+/−^ mice at postnatal days 30 (p30). As controls, this assay included mice with the following genetic backgrounds: rho+/−; Q344ter^rho−/−^; and rho−/−. From the various dark-reared mice, total RNA was isolated from individual retinas using the Trizol Reagent (Invitrogen Corp., Carlsbad, CA), and reverse transcription with random primers was performed to obtain cDNA. These cDNA products served as templates, in which a 250 bp fragment common to both WT and transgenic rod opsin transcript species - beginning at the 3′ of exon 4 and ending within exon 5 beyond the sites of mutagenesis – was amplified by PCR with the primers FACRhoEx4A (5′ GGTCATCTACATCATGTTGAACAAGC 3′) and mRh5 (5′ TGAGGGAGCCTGCATGACCTCATCC 3′). To label the amplification product, 10 µCi of α-^32^P dCTP (3000 Ci/mmol (GE Healthcare, Piscataway, NJ)) were added to the PCR buffer. These amplified products were precipitated, washed, resuspended in dH_2_O, and divided into two equal aliquots. One aliquot of 7.0 µl was digested with AvrII for Q344ter, and the other aliquot of equivalent volume was mock digested. AvrII digestion of transgene transcripts results in two fragments –122 bp and 128 bp. Both aliquots were then loaded in a 3% 3∶1 Nusieve agarose gel (ISC BioExpress, Kaysville, UT). The PCR fragments were transferred to Zetaprobe blotting membrane through capillary action with 0.4 N NaOH, and the intensities of the 250 bp radioactive bands were analyzed using the Storm 860 Phosphor Imager software (GE Healthcare). The Q344ter transgene expression level was determined as: 1– (intensity of AvrII-resistant radioactive PCR product in the digested aliquot divided by the intensity of the total intact radioactive PCR product in the undigested aliquot).

### Western blot analysis

Retinas were dissected from dark-reared mice at p30 under infrared illumination. Individual retinas were homogenized in buffer [80 mM Tris-HCl pH 8.0; 4 mM MgCl_2_; protease inhibitor cocktail (Roche Diagnostics, Indianapolis, IN); and 0.5 mM phenylmethylsulfonyl fluoride (PMSF)], and subsequently incubated with DNase I (Roche Diagnostics) for 30–45 min at room temperature. Equal volume of protein sample loading buffer (100 mM Tris, pH 6.8, 0.2 M dithiothreitol (DTT), 8% SDS, 20% glycerol, dash of bromophenol blue) was added, and the equivalent amounts of retina per sample (1/800) were loaded and separated in a 12% Tris-glycine polyacrylamide gel (Invitrogen Corp.). The protein samples then were transferred onto nitrocellulose membrane (Whatman Schleicher & Schuell) and were incubated with either the anti-N-terminal rhodopsin monoclonal antibody, R2-12N (1∶10,000) that recognizes residues 2–12 [19]; or the anti-C-terminal rhodopsin monoclonal antibody, 1D4 (1∶20,000) that recognizes residues 340–348 [20]. Goat anti-mouse IgG conjugated horseradish peroxidase (HRP; 1∶10,000; Vector Laboratories, Burlingame, CA) was used as the secondary antibody, and the signal was detected using the ECL system (GE Healthcare).

### Rhodopsin immunofluorescence

All mice were dark-reared and sacrificed at p30. Before enucleation, the superior pole for each mouse eye was cauterized for orientation. The mouse eye was first placed in fixative solution (4.0% paraformaldehyde, 0.5% glutaraldehyde in 0.1 M cacodylate buffer pH 7.2) for 5 min at room temperature (RT), after which the cornea was removed, and the lens was removed 10 min later. The eyecup was further fixed for 2 h and rinsed free of fixative with 0.1 M cacodylate buffer pH 7.2. The tissues were then infiltrated with 30% sucrose in 0.1 M cacodylate buffer for 14–18 hrs at 4°C, after which the eyecups were hemisected, embedded in Tissue Tek® O.C.T. (Sakura Kinetek U.S.A. Inc., Torrance, CA), and quickly frozen in liquid nitrogen. Ten micron frozen sections were obtained with a Jung CM 3000 cryostat machine (Leica Inc., Deerfield, IL). The retinal sections were incubated for 1 hour in blocking solution [2.0% BSA, 0.3% Triton X-100, and 2% goat serum in phosphate buffer saline (PBS)]. This blocking solution also served as the dilution solution for all involved antibodies. These sections were incubated with one of the following mAbs: R2-12N (1∶100) or 1D4 (1∶1000). After washing with blocking solution, the sections were incubated with a 1∶100 dilution of FITC-conjugated rabbit anti-mouse IgG (Vector Laboratories, Inc., Burlingame, CA). After a series of washing and a short fix (5 min in 4.0% paraformaldehyde in PBS), the sections were mounted with Vectashield (Vector Laboratories, Inc.), cover-slipped, and analyzed with an AxioPlan 2 imaging microscope (Carl Zeiss, Inc., Goettingen, Germany).

### Light and electron microscopy and retinal morphometry

The Q344termice and their negative control littermates were either dark-reared or dark-reared and exposed to five days of continuous light (3000 lux with undilated pupils) preceding their sacrifice. Eyecups were fixed and embedded in an epoxy resin as previously described [15], and sectioned at 1 µm or 60 nm thickness using an ultramicrotome (Leica Ultracut UCT, Leica Microsystems, Bannockburn, USA) for LM and EM, respectively. Electron micrographs were obtained on a JEOL JEM 2100 microscope. For retinal morphometry the eyecups were sectioned at or near the vertical meridian as determined by the optic nerve, and the outer nuclear layer thickness was measured based on a previously described method [24]. Briefly, retinal section was viewed by a microscope (40× objective) attached with a camera lucida; and measurements were taken with the aid of a graphics tablet (WACOM, Vancouver, WA) and the Axiovision LE Rel. 4.1. imaging software (Carl Zeiss Inc.). A stage micrometer (Klarmann Rulings, Litchfield, NH) was used for calibration. Each hemisphere - determined by the optic nerve - was divided into ten equal segments from the optic nerve to either the superior or inferior tip, and three measurements were taken and averaged for each segment. Due to the thinness of the outer nuclear layer at the optic nerve location, determination of the ten equal segments for each hemisphere excluded the first 100 µm from the optic nerve site. Statistically, when comparing sample populations to determine significant differences, t-tests were used with α = 0.05.

### Rhodopsin phosphorylation visualized by isoelectric focusing (IEF)

Dark-reared p30 Q344ter mice in rho+/− or rho−/− background and their respective transgene-negative littermates were either subjected to light-exposure by dilating their pupils and exposed to 3000 lux for 0.5 hr or kept in the dark. Retinas were collected and snap frozen in liquid nitrogen. Afterwards, all steps were performed under infrared illumination until the end of focusing run. The retinal samples were homogenized with a PT 1200 C polytron (Kinematica, Switzerland) in 400 µl homogenization buffer [25 mM Hepes pH 7.5, 100 mM EDTA, 50 mM NaF, 5 mM adenosine, 1 mM PMSF, and protease inhibitors (Roche Diagnostics)] and centrifuged at 19,000×g (4°C, 17 min). After washing with 10 mM Hepes pH 7.5, the pellet was resuspended in 1 ml regeneration buffer (10 mM Hepes pH 7.5, 0.1 mM EDTA, 50 mM NaF, 5 mM adenosine, 1 mM PMSF, 1 mM MgCl_2_, 2% BSA, protease inhibitors, and approx. 1000 pmol 11-cis retinal) and incubated overnight (O/N) at 4°C. The samples were spun down at 19,000×g and washed with 10 mM Hepes pH 7.5. The pellets were incubated in 50 µl or 100 µl solubilization buffer [20 mM Hepes pH 7.5, 0.1 mM EDTA, 50 mM NaF, 5 mM adenosine, 1 mM PMSF, 1 mM MgCl_2_, 10 mM NaCl, 1% dodecyl-maltoside, 1 mM dithiothreitol, protease inhibitors] for 3–24 hrs at 4°C. Glycerol was added to the solubilized pellet samples, which were loaded onto an acrylamide gel [5% acrylamide, 0.01% DM, 13.33% glycerol, 3.8% Pharmalyte pH 2.5–5 (GE Healthcare), 2.53% Pharmalyte pH 5–8 (GE Healthcare), catalyzed by ammonium persulfate and TEMED]. The sample amounts (fraction of a retina) are as follows: rho+/− (1/20); Q344ter^rho+/−^ (1/10); rho−/− and Q344ter^rho−/−^ (1/5). The samples were run at a constant 23 W, with 0.04 M glutamic acid as the anode solution and 1.0 M NaOH as the cathode solution, on a Pharmacia Flat Bed Apparatus FBE300 (GE Healthcare) at 10°C for 2 h. Afterwards, the proteins were transferred onto nitrocellulose membrane by capillary action with PBS. The membranes were subjected to immunoblotting analysis with 1D4 and R2-12N or 4D2 monoclonal antibodies.

### [^35^S]GTPγS (Guanosine 5′-O-(γ-thio) triphosphate) in situ loading assay

Q344ter^rho+/−^, Q344ter^rho+/−Tra−/−^ and their transgene-negative littermate controls were dark-reared and sacrificed at P28–31. Unless otherwise stated, all work, including cryosectioning, was performed under infrared light. After removing the cornea and lens, the eyecup was embedded in 3% low-melting agarose (Sigma-Aldrich, St. Louis, MO) dissolved in Ames's like solution [10 mM HEPES pH 7.4, 2 mM NaHCO_3_, 110 mM NaCl, 2.5 mM KCl, 1.0 ml CaCl_2_, 1.6 mM MgCl_2_, 10 mM glucose). The agarose-embedded eyecups were placed in Tissue Tek® O.C.T. compound (Sakura Kinetek U.S.A. Inc., Torrance, CA) and frozen in liquid nitrogen. The frozen tissue were transferred to −20°C and sectioned (10 µm) with a Jung CM 3000 cryostat machine (Leica Inc., Deerfield, IL).

The [^35^S]GTPγS autoradiography was based on previously described protocols with modifications [68], [69]. After allowing the frozen sections to reach room temperature, they were incubated for 10 min in ROS buffer (20 mM HEPES pH 7.4, 120 mM KCl, 5 mM MgCl_2_, 1 mM dithiothreitol, 100 µM phenylmethylsulfonyl fluoride) to remove the surrounding mounting medium. The tissue samples were then equilibrated for 1 hour in preincubation buffer [100 µm guanosine –5′-O- diphosphate (GDP, disodium salt form, MP Biomedicals, Irvine, CA), 2 mM β-nicotinamide adenine dinucleotide phosphate (NADPH, reduced tetra(cyclohexylammonium) salt form, Sigma-Aldrich) in ROS buffer]. The tissue sections were then incubated with the “hot” reaction buffer [100 nM GTPγS (Roche Diagnostics, Indianapolis, IN), 20 nM [^35^S]GTPγS (1000 Ci/mmol; GE Healthcare, Piscataway, NJ) in preincubation buffer] and either remained in darkness or exposed to light (3000 lux) for 20 min. Non-specific binding is measured with “cold-excess” reaction buffer (20 µM GTPγS, 20 nM [^35^S]GTPγS in preincubation buffer) for 20 min. After incubation, all tissue samples were transferred back to the dark and washed 4×5 min with ROS buffer and 1×30 sec with dH_2_O. The sections were then air dried for 20 min and submerged in autoradiography emulsion NTB (Eastman Kodak Co., Rochester, NY), and allowed to dry (30 min). Afterwards, the sample slides were stored in a light-tight container at −80°C for 3 days.

To develop the film, the sample slides were submerged for 3.5 min in Developer-19 solution (Eastman Kodak Co.), rinsed with dH_2_O, and submerged for 5 min in Kodak fixer solution. After drying the slides for 20 minutes, the samples were stained with 0.4% Toluidine Blue O solution (Sigma-Aldrich) and washed with phosphate buffer saline (PBS). This staining of nuclei provided general orientation of the retinal cell layers. The sections were dehydrated in graded alcohol: 1×10 min with 50%, 70%, 90% ethanol, 2×10 min with 100% ethanol, and finally 2×10 min xylene. The sections were viewed and photographed with an AxioPlan 2 imaging system (Carl Zeiss, Inc.).
